# The Impact of COVID-19 Lockdown on Agility, Explosive Power, and Speed-Endurance Capacity in Youth Soccer Players

**DOI:** 10.3390/ijerph18189604

**Published:** 2021-09-12

**Authors:** József Márton Pucsok, Miklós Kovács, Gergely Ráthonyi, Balázs Pocsai, László Balogh

**Affiliations:** 1 Institute of Sport Sciences, University of Debrecen, 4032 Debrecen, Hungary; koxi11@mailbox.unideb.hu (M.K.); balogh.laszlo@sport.unideb.hu (L.B.); 2 Faculty of Economics and Business, Institute of Applied Informatics and Logistics, University of Debrecen, 4032 Debrecen, Hungary; rathonyi.gergely@econ.unideb.hu; 3 Sportdiagnostic and Therapeutic Center, 4032 Debrecen, Hungary; pocsai.balazs@setcenter.hu

**Keywords:** soccer, athletic performance, physical fitness, performance testing, SpeedCourt System

## Abstract

Our goal was to assess agility, explosive power, and speed-endurance capacity by implementing noninvasive procedures and sport-specific tests. We hypothesized that agility, speed, explosive power, and speed-endurance capacity might be maintained or increased by an individualized home-based training program. Eleven adolescent athletes participated in our study; they executed three tests before the coronavirus outbreak and 13 weeks later, after the pandemic curfew. We used the SpeedCourt System to assess the sport-specific speed and agility parameters and monitor speed-endurance capacity. We conducted the first measurement at the end of the preparatory period, on 28 February 2020. The second session consisted of 4 weeks of regular training and 9 weeks of individual, home-based activities. Compared to the first (pre-pandemic) testing session, our participants demonstrated a significantly improved capacity of the lower limbs’ explosive strength after completing the home-based exercise routine, compared to the first (pre-pandemic) testing session. We found that agility, speed, and explosive power might be maintained at the same level under home-based conditions. We found that it was challenging for the participants to increase their “pre-pandemic” endurance capacities.

## 1. Introduction

Modern-day sports science is based on achieving the highest possible training intensity, which would simulate the situations that tend to occur in a game. Alternating intensities is one of the critical elements of an efficient training routine. At the same time, it is necessary to prepare athletes to reduce the risk of injuries [1].

High-intensity intermittent movements primarily characterize soccer. High-speed runs in a game are standard for a top-level player to perform 17–30 shorter or longer submaximal sprints [2]. It is a common misconception that repetitive sprint ability is one of the utmost important aspects of soccer training [2,3]. International elite level players are characterized by sprints faster than 25 km/h [2]. Realistically, athletes may not achieve these high-speed movements in every second of the game. Researchers found an average gap of 5 min and 12 s between each sprint by examining the German National Team’s speed performance [3]. Similar results have been reported by previous studies [4,5,6,7,8]. High-speed activities dominate the sport; however, maximal high-speed sprints reaching 30–35 km/h occur relatively infrequently, only around two bouts in a game [4].

An important parameter is the number of so-called micro-movements, i.e., accelerations, decelerations, jumps, and direction changes. Bompa and Carrera [9] described this phenomenon in their 2014 study which defines the world’s most popular team sport, soccer, as a sport requiring high technical and physical readiness. In ball sports, especially soccer, successful athletes are characterized by high-level explosive power, speed, agility, and special endurance. 

The more the athlete can repeat these high-energy movements, the higher their performance [10]. Taylor and his colleagues [2] compared different team sports according to the intervals when there is a change of activity. When we examine the total distances traveled throughout a game, surprisingly, soccer players may cover similar distances as in other sports such as handball and basketball, played in a significantly smaller area [2]. 

It is challenging to maintain the pace of these multi-directional movements throughout the entire game, for 90 min or even longer. Therefore, speed-endurance capacity is one of the core skills in modern soccer [11]. Indirect measurement of the endurance capacity in a field-based environment is always challenging. Commonly used post-exercise heart rate recovery (HRR) is a reliable indicator of cardiovascular fitness. HRR is readily obtainable and may be associated with other aerobic endurance variables such as maximal oxygen uptake [12]. 

Agility and change of direction speed (COD) are commonly assessed in team sports. There are a number of field tests to evaluate specific skills; the Illinois Agility Test and the 505 Test are widely used by team sport professionals [13,14]. Although they are reliable methods to assess specific skills such as agility and COD, they cannot entirely mimic the particular needs of a given team sport [15]. SpeedCourt is a widely adjustable measuring device able to mimic the specific demands of the given sport (soccer). First, Düking et al. [15] investigated the reliability, usefulness, and validity of the SpeedCourt System. The researchers examined total sprint time (TT) and time to change of direction (TCD). They found that regarding TT, SpeedCourt is a reliable and valid measurement device to assess performance. 

## 2. Purpose of the Study

The primary aim of the study was to investigate the effect of the COVID-19 lockdown on athletic performance in youth soccer players. The secondary purpose of our study was to examine the efficiency of an individual, home-based training routine in maintaining and developing specific conditioning skills. We hypothesized that athletes would maintain or increase their pre-pandemic fitness level through three carefully planned and executed workouts a week. We suggested that the strength of the lower limb, step frequency, and speed-endurance will demonstrate soccer players’ current level of skills. 

## 3. Material and Methods

### 3.1. Study Participants

Eleven male adolescent soccer players participated in our study. To ensure homogeneity of the sample, we decided to exclude the goalkeeper from the analysis due to his differences in body composition and physical abilities [16]. Their average age, height, and weight were 16.7 ± 0.6 years, 175.7 ± 6.2 cm, and 67.6 ± 7.6 kg. Mean body fat values were 11.11 ± 3.36 % for all participants. We conducted a body composition analysis utilizing a Tanita Pro BC-1000 device (Tanita Europe BV, Amsterdam, The Netherlands). Out of eleven field players, three strikers, five midfielders, and four defenders participated in our investigation. They represented the elite level of their sport, with 8 ± 2 h of regular practice a week. The participants had a mean of 7.1 years of experience in playing soccer. All participants reported no injuries at the time of the investigation. All examinations involving human subjects were approved by the Hungarian Ethical Committee “Országos Tisztifőorvos Feladatokért Felelős Helyettes Államtitkárság, Egészségügyi Igazgatási Főosztály” Office of the Surgeon General, Department of General Health Issues (approval number: 15117-4/2018/EÜIG). All measurements were conducted based on the Declaration of Helsinki; additionally, informed consent was signed by all participants and their guardians accordingly.

### 3.2. Home-Based Training Routine

After the first tests, the participants continued their regular practice with three sessions a week. However, it was subsequently forbidden to train on a soccer field for individuals and teams as well. Spring 2020 was the worst period in terms of the COVID-19 pandemic in Hungary. In the first week of February 2020, there were 8909 confirmed cases in Hungary; by the end of March, there was a sharp increase of 64.36 % in the number of confirmed cases [17]. As a result, we prescribed a series of exercises for soccer players at home for 13 weeks, according to the following methodology:

Each training session’s duration was 60 min. The participants performed 12 min of a warm-up session, including a mobilization block and dynamic stretching. Finally, a 6 min cool-down (easy jogging and stretching) session was performed. Participants monitored intensity individually by using the modified Rate of Perceived Exertion (RPE) scale. 

Training session one: The main objective of this session was to improve endurance. On the first training day of the week, athletes performed regular outdoor running for 12 min, repeated three times. We provided 2 min of recovery time. We set intensities at seven on the RPE scale. After each training session, we provided 48 h of regeneration time. 

Training session two: On the second training day of the week, strength-endurance training was performed (both upper and lower limbs). Athletes performed twelve exercises, repeated three times with their body weight. We divided the entire training routine into three (5-4-4 weeks) periods. We incorporated a load/rest ratio of 25–35 s, 30–30 s, and 35–25 s, respectively. Athletes performed 2.5 min of passive rest at the end of each set, while 48 h of rest were provided at the end of each session. The training sessions of the uneven weeks consisted of four sets of exercises: Copenhagen holds (adductor plank) on both sides, push-ups, bicycle crunches, Nordic hamstrings and squats (sets 1 and 2), shoulder push-ups, hyperextensions on the ground, single-leg glute bridges on both sides, bench dips and inverted rows (sets 3 and 4). The training sessions during the even weeks also incorporated four sets of exercises: alternate leg lunges, step-ups, decline push-ups, heel touches, bird dogs, calf raises (sets 1 and 2); forearm to push-up planks, superman exercises, lateral squats, sit-ups, incline narrow push-ups and chin-ups (sets 3 and 4).

**Figure 1:**
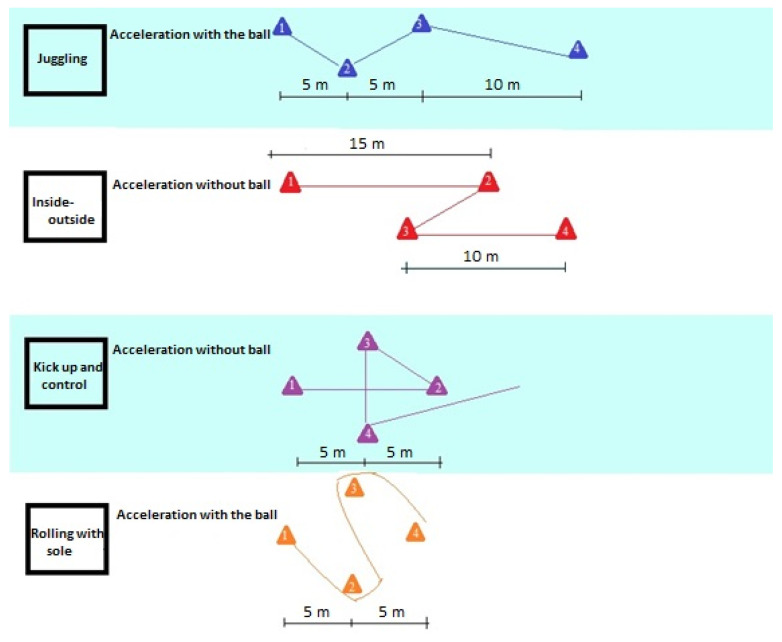
Ball control and acceleration routine. Notes: (1–4) Sequence of the exercises.

Training session three: This session was based on multi-directional movements and jumps. Training session “A” was performed in uneven weeks, including four multi-directional runs and micro-movements. In contrast, session “B” was performed in even weeks and included four plyometric jumps to improve reactive strength. We designed the exercises for the participants to perform even in a relatively small 5 × 5 m^2^ area (room). During session “A”, participants performed rapid, explosive (2–4 m) accelerations followed by intense changes of direction (3–4 sets of each), and at the end, there were 2–3 m linear accelerations. Session “A” included various ball control drills such as juggling, inside-outside touches, kick-ups, and control, rolling with the sole. Agility and acceleration drills: zig-zag runs (2 × 5 m), linear accelerations (10 and 15 m), and cross-runs (3 × 5 m) were also completed. Numbered triangles defined each pathway in numerical order (Figure 1). 

**Figure 2:**
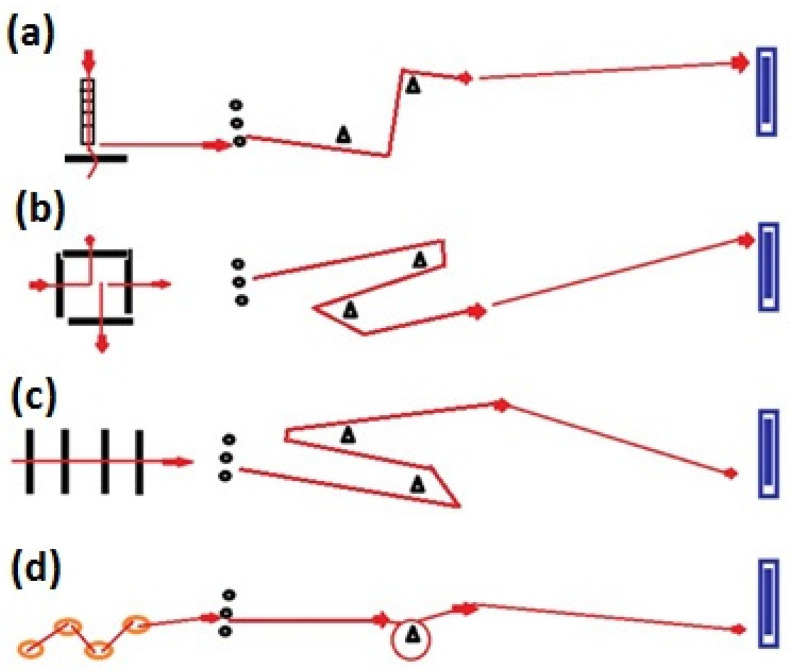
Agility and explosive strength routine. Notes: (**a**) first exercise routine; (**b**) second exercise routine; (**c**) third exercise routine; (**d**) fourth exercise routine.

During session “B”, they completed plyometric activities and vertical jumps over 20–40 cm high household obstacles. At the end of each repetition, they executed 2–3 m linear accelerations. We designed four sets of exercise routines. The first exercise routine (a) included ladder runs, forward-backward jumps, zig-zag runs, and acceleration with the ball. The second exercise routine (b) incorporated forward-leftward-rightward-rightward-leftward-forward jumps, zig-zag runs with the ball, and acceleration with the ball. The third exercise routine (c) included repeated forward jumps (four times), zig-zag runs with the ball, and acceleration. The fourth exercise routine (d) contained lateral hops (four times) and acceleration with the ball and around the cone (Figure 2). Participants executed each exercise for 10–15 s at maximum intensity (90–100%), repeated six times. We provided 45–50 s of rest between each repetition. Between sets, there was 4 min of passive rest. After the training session, we provided 72 h of rest for the athletes.

### 3.3. Measurement Procedures

We conducted the measurements at the end of the preparation period on 28 February 2020 and 13 weeks after the COVID-19 spring outbreak. Three tests and an HRR measurement were performed for all participants. Before the tests, the participants completed a 5-minute individual warm-up. All participants were familiarized with the SpeedCourt System before starting the measurement process. They were adequately hydrated and refrained from consuming any food for at least an hour beforehand. All tests were conducted between 9 and 12 am. Participants executed all tests three times, with 30 s and 1 min of rest. We assigned the participants into four groups; the groups had 3, 3, 3, and 2 members. First, group one completed the Modified Quick Feet Test (MQFT) to eliminate fatigues’ deteriorating effect on the speed performance. Participants performed maximal stationery running for 5 s. Next, the soccer players executed the Countermovement Jump Test (CMJ) to assess the lower limbs’ power. They completed both tests on the central contact plate. 

Finally, we conducted a 60-meter chasing test (60 m CT) to monitor repetitive micro-movements capability. The 60 m CT was designed and developed by our research team and assessed speed-endurance based on HRR data taken during the 1 min rest periods between them.

**Figure 3:**
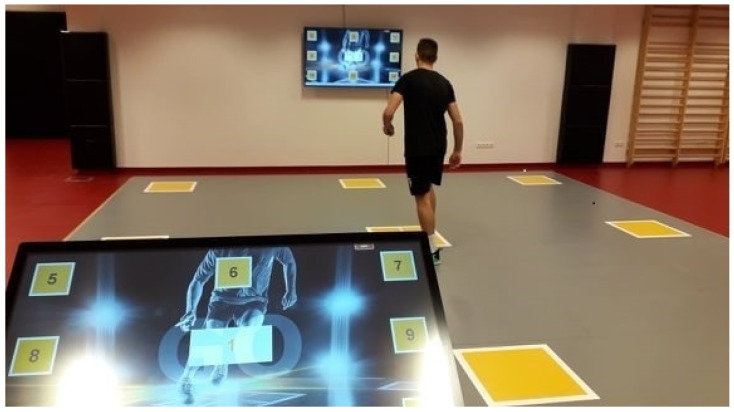
The SpeedCourt System.

Participants completed all tests on SpeedCourt^®^ 550 Q12 (Global Speed GmbH, Hemsbach, Germany) measuring device at the Sportdiagnostic and Therapeutic Center, Debrecen, Hungary. The SpeedCourt’s total area is 7 × 7 m^2^, equipped with nine contact plates (sensors) positioned 3.54 m apart (Figure 3).

**Figure 4:**
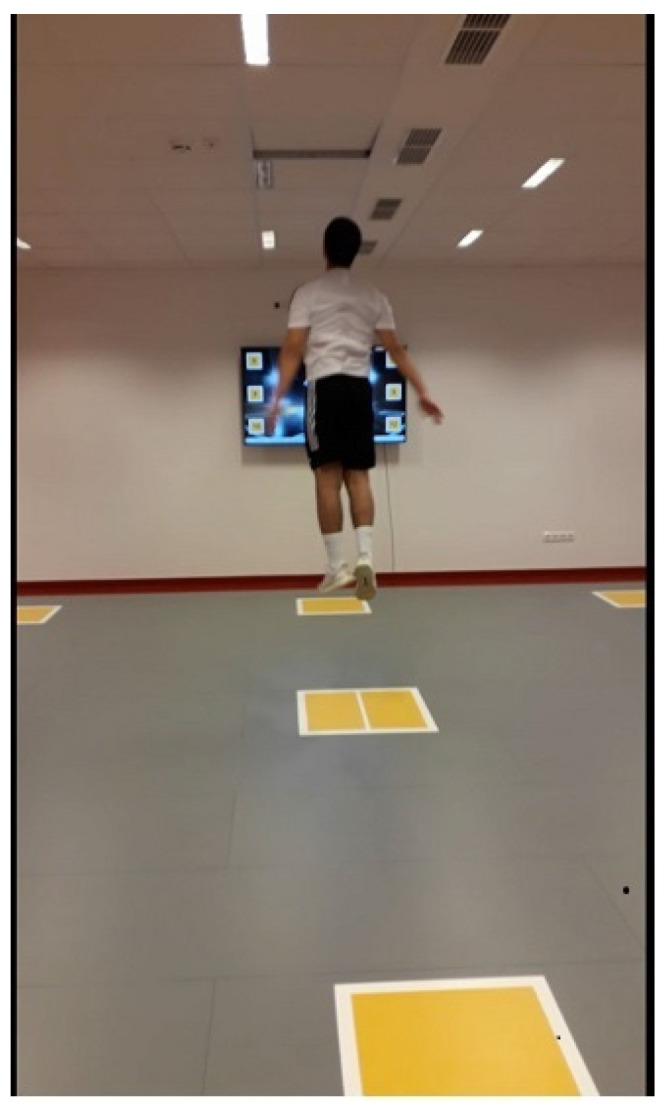
Countermovement Jump Test.

We measured the lower limb’s explosive strength (Figure 4) by the CMJ. The device calculated the vertical height of each jump according to the duration of the non-contact phase. Participants completed the three experiments with a passive rest period of 30 s [18]. They executed the vertical jump according to the natural movement patterns of soccer. The participants initiated the vertical jump with an arm swing from a slight knee bending position [19].

#### 3.3.1. Step Frequency

The participants performed a maximum number of steps with alternating feet (tappings) in 5 s. The participants completed the MQFT on the central contact panel; they initiated the test procedure in motion and the final 3 s were recorded. The athletes had to execute three trials with 30 s of rest between them. The best result of the three trials was later analyzed.

#### 3.3.2. Reactive Agility Test

**Figure 5:**
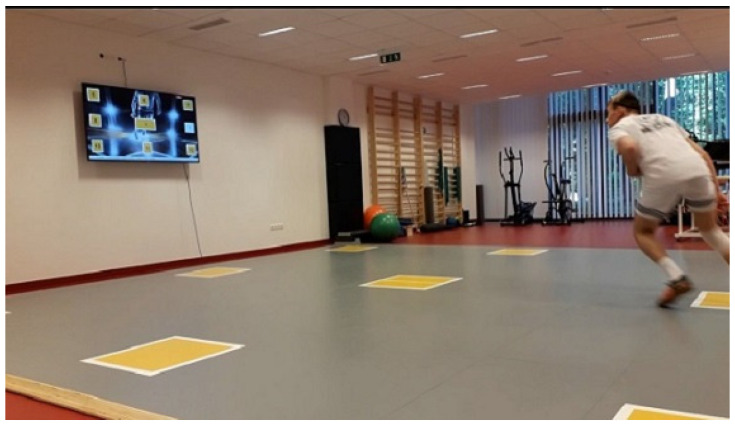
60 m chasing test.

The 7 m^2^ floor area of the SpeedCourt track was perfectly adequate for our research purposes [14,15]. We conducted the 60 m CT (Figure 5), developed specifically for soccer players by our research team. Athletes initiated the test procedure on a computer-controlled signal. They completed a preset route, touching the nodes with one foot in 3 × 3 formation as quickly as possible. Participants were guided by a digital display instructing which number-coded contact plate to move. Each athlete performed the test three times, with 1 min of rest. They performed the 60 m CT in pairs in a rotating system. For the first time, they completed the test one after the other. The essence of the trial was to complete the 60 m distance as fast as possible. If the contact plate detected no contact, we instructed the athlete to return to their original position and perform the touch again. This type of spatial disorientation primarily characterized only the first attempts of the participants. While conducting the three trials, we provided 1-min recovery time; meanwhile, the next pair was prepared with a solo warm-up. 

#### 3.3.3. Post-Exercise Heart Rate Recovery

**Figure 6:**
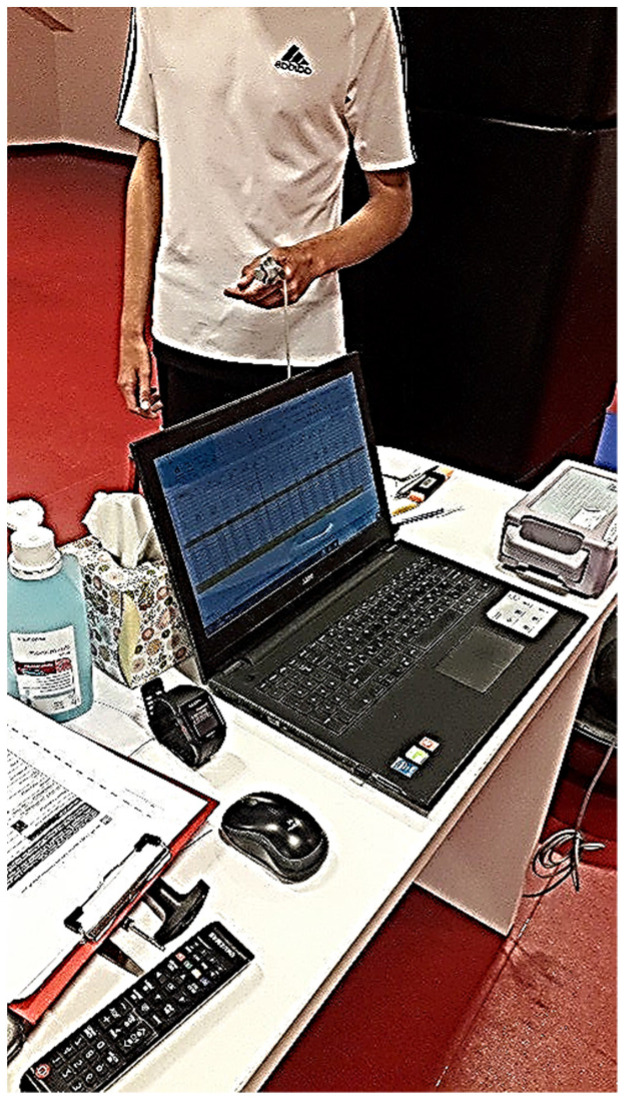
Heart rate measurement device.

During the 60 m CT, we used a Nellcor OxiMax N-560 pulse oximeter device (Figure 6) (Medtronic, Minneapolis, MN, USA) to record the HRR values. We conducted the first measurement immediately after (within 5 s) the test. We monitored the athletes right next to the SpeedCourt testing area in a stationary position to avoid any errors in the measurement process. We calculated the HRR value as the difference between the post-exercise and the values measured at the end of the 1 min rest period. 

### 3.4. Statistical Analysis

All analysis was performed using the SPSS for Windows (version 21) software (StatSoft. Inc., Tulsa, OK, USA). Mean values and standard deviations were calculated for each test. First, we conducted a test for normal distribution. Because the sample size was lower than thirty, we utilized the Shapiro–Wilk test (Table 1). After we confirmed normal distribution, we either performed the paired *t*-test or if the *p*-value was lower than 0.05 we performed the Wilcoxon test. We also calculated the effect size to assess the magnitude of differences between group means [20]. We used the conventional effect size proposed by Cohen [20] for the analysis, where 0.2, 0.5, and 0.8 were treated as small, medium, and large, respectively. For all analyses, the level of significance was at *p* < 0.05. 

## 4. Results

### 4.1. Lower Limb Explosive Strength

Eleven athletes performed the CMJ to assess the explosive strength of the lower limb. We compared mean values and the best and the worst attempts before and after the pandemic. We considered differences between the trials lower than 1 cm as equals. First, we analyzed the mean values (43.23 ± 5.88 cm; 44.95 ± 7.32 cm); the difference was statistically significant (*z* = −2.31; ES = 0.71; *p* = 0.03) (Table 2). Seven participants improved their results, three results remained unaltered, and only one demonstrated a moderately decreased jumping performance compared to the first testing session. Five athletes could outperform their individual bests, four performed equally, and two athletes underperformed compared to the pre-pandemic results.

### 4.2. Step Frequency

We measured the step frequency of the participants (*n* = 11) using the MQFT. The pre-and post-pandemic mean values demonstrated improvement for six participants, three performed equally, and two underperformed (31.39 ± 3.50 steps; 33.03 ± 3.71 steps). We treated one step difference between the test trials as a similar result. However, the difference was statistically not significant (*z* = −1.47; ES = 0.54; *p* = 0.10) (Table 2). Six athletes improved their personal bests, two maintained their pre-pandemic performance level, and three of them demonstrated a decrease in performance.

### 4.3. Reactive Agility

We conducted the 60 m CT to assess the agility of the participants (*n* = 11). During the evaluation process, two trials were equal within a 0.5 sec difference. We compared pre- and post-pandemic mean values (24.11 ± 1.26 s; 27.11 ± 1.50 s); the difference was not statistically significant (*z* = −0.08, ES = 0.15; *p* = 0.62) (Table 2). Out of eleven athletes, three demonstrated an increase, three showed a decreased performance, and five reached identical results. Only one athlete could improve his individual best; five of them underperformed and five demonstrated identical results. 

### 4.4. Post-Exercise Heart Rate Recovery

We assessed speed-endurance capacity [21] by the HRR values measured after completing the 60 m CT. We treated two beats/min or marginal differences as identical. First, we analyzed the mean values of the pre- and post-pandemic trial sessions (26.08 ± 5.82 beats/min; 23.52 ± 8.54 beats/min); the difference was not statistically significant (*z* = −0.84; ES = 0.33; *p* = 0.29) (Table 2). Seven out of eleven participants demonstrated a deteriorated recovery capacity and four indicated improved recovery; we also observed a deteriorating tendency during the post-pandemic measurements in HRR values between the first and final attempts. Eight out of the eleven participants demonstrated high values and decreased recovery capacity; in one athlete, the recovery capacity remained unaltered, and only two could improve. During the pre-pandemic test session, we observed a somewhat different tendency. Six athletes’ recovery capacity decreased, four improved their results, and the ability remained unaltered in one case.

## 5. Discussion

One of our main findings is that young male soccer players may be able to increase their physical fitness level under home-based conditions. We measured a significant improvement in lower limbs’ explosive strength in the second (post-pandemic) testing session. We found no significant difference in step frequency and reactive agility. Our athletes could not perform the necessary volume of micromovements (jumps, hops, accelerations, decelerations, and change of directions) to improve their agility performance. Due to the lack of locomotor motion, we measured a moderately impaired speed-endurance capacity at the post-pandemic testing session.

Outbreak of the COVID-19 pandemic resulted in an unprecedented situation in the first half of 2020. Athletes had to temporarily pause their regular training sessions. The athletes performed individual workouts. Team sports require high mental preparedness to execute any tactical drills. Individual exercises may effectively improve only conditioning skills such as strength, agility, flexibility, and endurance; tactical skills may start to deteriorate without team practices. Rapid turns and high-frequency movements primarily characterize ball sports. We aimed to implement SpeedCourt test protocols to mimic the specific characteristics of soccer [22]. Since we recorded the total times of the trials, our study may be defined as reliable and valid in agility and change of direction measures [15]. Our study focused on sport-specific skills such as vertical jump height, step frequency, and agility; these skills have been widely assessed in ball sports [14,18,22,23]. Most of the investigations in the past utilized various field tests measuring movement frequency, COD, and agility [23,24,25,26]. The introduction of the SpeedCourt System revolutionized the assessment of multi-directional movements and maneuvers [15,22]. 

Other researchers also investigated the effect of COVID-19 lockdowns on physical performance among elite-level futsal and soccer players [27,28]. A recent study by Spyrou et al. [28] examining 10 m sprint, horizontal and vertical jump performance suggested that even long-term (70 days) reduced training did not result in a significant decline in vertical jump performance. On the other hand, Rampini et al. [27] measured a significant decline in vertical jump performance after completing a home-based training routine. Our studied population demonstrated significantly higher vertical jump height values at the post-pandemic testing session. One of the significant differences between our and previous studies [27,28] is that we implemented a preplanned home-based training routine. We suggest that without a carefully designed home-based exercise routine, vertical jump performance cannot be improved.

MQFT is widely used to assess the step frequency of soccer players [29]. Step frequency is strongly correlated with running performance, including short-distance sprinting ability in young soccer players [30]. Other researchers also found a significant relationship between step frequency and short linear sprinting ability or acceleration [15,29]. We found no related studies examining the effect of COVID-19 lockdowns on step frequency in soccer players. Our participants demonstrated an improvement in step frequency; however, the differences were not significant. 

Reactive ability is one of the critical components in ball sport performance [22]. Born et al. [14] implemented a longitudinal study design, assigning participants into two groups. They investigated the effect of three-week multi-directional sprint training on speed and reactive agility. Six sessions of SpeedCourt training provided additional benefit in terms of COD, but not on vertical jump height and 20 m sprint time [15]. Matlák et al. [22] investigated the relationship between COD and reactive agility among soccer players. They found no significant relationship between COD and reactive running tasks. Reactive agility tests are performed in a randomized way; in contrast, COD tests are preplanned. During the agility test, continuous external visual stimuli are provided by the SpeedCourt System so that participants may improve their sport-specific skills more effectively. These findings further supported the reliability of our study design utilizing the SpeedCourt System as a testing device. 

The post-exercise heart rate recovery capacity was moderately impaired at the post-pandemic testing session. The home-based training routine could not provide the necessary load and intensity to improve HRR capacity. HRR value strongly corresponds to the level of aerobic fitness; thus, endurance capacity is impaired too [31]. We suggest that home-based training could not provide a sufficient amount of locomotor movements to maintain speed-endurance capacity. However, a recent study [32] questions the reliability of HRR as a predictor of high-intensity performance (cycling) readiness. Further investigations need to be conducted on this topic, involving multi-directional movements as well. 

### 5.1. Limitations of Our Study

A relatively low number of soccer players participated in our study. A more robust sample size would result in more reliable conclusions. We were aware that training intensities would be more effectively monitored by digital applications (Polar watches, etc.) instead of the RPE scale. We realized that a vertical jump without an arm swing would more realistically assess the explosive power of the lower limbs. However, we assumed that incorporating an additional arm swing is more soccer-specific [33].

### 5.2. Practical Applications

Our result underscored the vital role of external stimuli in soccer-specific training and testing. The improvement of reactive and decision-making abilities is vital in ball sports. Soccer players may benefit from the prolonged training stimuli (13 versus 3 weeks) provided by the SpeedCourt device. However, the proper duration of SpeedCourt training depends on the participants’ age, biological maturation, fitness status, and type of activity performed [23]. Further research is necessary to support these hypotheses.

## 6. Conclusions

This study supported our prior hypothesis that adolescent male soccer players may be able to maintain or increase their pre-pandemic fitness level under home-based conditions. The explosive strength of the lower limb significantly improved compared to the first (pre-pandemic) testing session. We may suggest that home-based training sessions could not mimic or substitute the number of micromovements (jumps, hops, accelerations, decelerations and change of directions) necessary for high-quality agility performance. However, it is essential to mention that appropriate spatial orientation and coordination are also needed to complete the tests [22]. We may conclude that the participants could maintain the overall quality of some performance variables such as vertical jumping performance, step frequency, or agility. We experienced significant improvements only in the level of lower limb explosive strength; the calculated effect size value (0.71) further supported our conclusions. The speed-endurance capacity of the athletes was moderately impaired after completion of the 13-week home-based training routine. We suggest that home-based training sessions replace, to some extent, regular (outdoor) training. However, the limited number of locomotor movements such as running impairs speed-endurance capacity, one of modern-day soccer’s vital performance components. We found only a limited number of studies using the SpeedCourt System in soccer players. As of now, we found no related studies investigating the effect of home-based training on sport-specific skills in youth soccer players. Our study, incorporating a home-based training routine and SpeedCourt System as a testing device, is novel this way.

## Figures and Tables

**Table 1:** Test of normality.

Shapiro–Wilk Test			
Tests	Statistic	*df*	Sig.
60 m CT pre	0.94	11	0.62
60 m CT post	0.95	11	0.72
HRR pre	0.83	11	0.02 *
HRR post	0.88	11	0.12
CMJ pre	0.94	11	0.53
CMJ post	0.84	11	0.03 *
MQFT pre	0.93	11	0.46
MQFT post	0.83	11	0.02 *

* *p* < 0.05. Notes: CT, chasing test; HRR, heart rate recovery; CMJ, Countermovement Jump Test; MQFT, Modified Quick Feet Test.

**Table 2:** Statistical analyses for all test procedures.

Pairs	Tests	Paired *t*-Test		Effect Size	Wilcoxon Test	
		*t*	*p*	Cohen’s *d*	*z*	*p*
Pair 1	60 m CT pre–60 m CT post	−0.51	0.62	0.15	−0.08	0.92
Pair 2	HRR pre–HRR post	1.10	0.29	0.33	−0.84	0.39
Pair 3	CMJ pre–CMJ post	−2.38	0.03 *	0.71	−2.31	0.02 *
Pair 4	MQFT pre–MQFT post	−1.79	0.10	0.54	−1.47	0.13

* *p* < 0.05.

## Data Availability

Data sets available upon request (contact contributing author).
